# Are Autochthonous Bacteria of Desert Root Environments Capable of Increasing Crop Tolerance to Saline Stress?

**DOI:** 10.3390/plants15060892

**Published:** 2026-03-13

**Authors:** Vincenzo Aurilia, Alessandra Ruggiero, Cuihua Huang, Jing Pan, Xian Xue, Anna Tedeschi

**Affiliations:** 1 Institute for Agriculture and Forestry Systems in the Mediterranean, National Research Council of Italy, P.le Enrico Fermi, 1, Località Porto Granatello, Portici, 80055 Naples, Italy; vincenzo.aurilia@cnr.it; 2 Institute of Biosciences and Bioresources (IBBR), National Research Council of Italy, Research Division Portici, Via Università 133, Portici, 80055 Naples, Italy; alessandra.ruggiero@cnr.it; 3 Drylands Salinization Research Station, Northwest Institute of Eco-Environment and Resources, Chinese Academy of Sciences, 320 West Donggang Road, Lanzhou 730000, China; hch@lzb.ac.cn (C.H.); panjing@lzb.ac.cn (J.P.); xianxue@lzb.ac.cn (X.X.)

**Keywords:** salinity, plant growth-promoting bacteria, biomass, WUE

## Abstract

Plant growth-promoting bacteria (PGPB) could be an alternative for alleviating salinity problems in different plants grown under salinity conditions. The study aimed to evaluate the ability of a bacterial consortium, isolated from the rhizosphere of the species *Lycium chinense* (LC), with the common name Goji, to alleviate the effect of salt stress on the crop response of two treated *Lycium* species. The bacterial consortium was applied in a pot experiment under controlled conditions to evaluate whether the consortium had any plant growth promoting effect on plants. Specifically two *Lycium* species *Lycium chinense* (LC) and *Lycium barbarum* (LB) were grown under saline (Ts) and not saline irrigation (Tc), and with (I) or without (NI) consortium inoculation. Inoculation of LB under salinity stress (Ts) significantly improved the leaf area compared to the uninoculated treatment (NI), i.e., 88.8 cm^2^ LB-I-Ts vs. 48.5 cm^2^ LB-NI-Ts. In LC, no significant difference was reported in the leaf area. Under salinity stress (Ts), the dry matter for both *Lycium* species significantly increased when inoculation occurred. The I treatment led to a higher WUE under the Ts treatment in both LC and LB. The inoculation (I) had a significant effect on the RWC. It was significantly higher under the I than the NI treatment, i.e., 82.5% vs. 77.0% at *p* ≤ 1%. The analysis of our results highlights that inoculation with the bacterial consortium has a substantially beneficial effect on plants in the presence of salt stress compared to non-saline plants. Furthermore, among the two *Lycium* species, the beneficial effect of inoculation with PGPB, in conditions of salt stress, is more evident in LB than in LC. Although the detailed mechanism underlying the PGPB activity was not elucidated, the results obtained support the potential beneficial use of soil bacterial species adapted to harsh conditions in the development of productive agricultural systems in saline environments.

## 1. Introduction

Soil salinization is a major constraint on agricultural productivity worldwide [1]. According to FAO estimates, approximately 1.38 billion hectares of land, about 10.7% of the global land, are affected by salinity [2]. In arid and semi-arid regions, where evapotranspiration exceeds precipitation, salt accumulation can severely reduce crop yields, in some cases by up to 70%. With ongoing climate warming and increasing aridity, the extent and intensity of salt-affected soils are expected to further expand.

Beyond its global extent, salinity exerts profound impacts on soil structure and plant physiological processes. Arid and semi-arid regions, already characterized by fragile soil systems and water scarcity, are particularly vulnerable. Salinization degrades soil structure, reducing porosity and altering water storage and movement [3,4], while climate warming accelerates evapotranspiration and salt accumulation, reinforcing land degradation processes. At the plant level, excess salts impose osmotic stress that limits water uptake, leading to physiological drought. Ionic toxicity and nutrient imbalance further disrupt metabolic processes [5], triggering oxidative stress and impairing photosynthesis, ultimately resulting in reduced biomass accumulation and crop productivity. In this context, developing sustainable strategies to mitigate salt stress is essential for maintaining agricultural production in dryland ecosystems.

To counteract salinity, various management approaches have been developed, including engineering measures such as salt leaching through open or subsurface drainage systems, irrigation management strategies, chemical soil amendments, the use of salt-tolerant species, and practices that reduce surface evaporation. These measures may be implemented individually or in combination. However, many conventional strategies require substantial freshwater input or long-term soil modification, limiting their sustainability in water-scarce environments. Consequently, increasing attention has been directed toward environmentally friendly and nature-based approaches that enhance plant adaptive capacity through biological interactions.

In recent decades, plant–microbe interactions have emerged as a promising avenue for improving crop performance under abiotic stress. The rhizosphere of plants growing in saline and arid environments hosts diverse microbial communities capable of contributing to plant survival under harsh conditions. Through root exudates, plants recruit beneficial microorganisms that participate in nutrient cycling, stress signaling, and soil structural stabilization [6,7,8]. Identifying and utilizing root-associated microorganisms to promote sustainable crop production under abiotic and biotic stresses has therefore become a major research focus.

Among plant-associated microorganisms, two main categories of beneficial bacteria can be distinguished: symbiotic bacteria forming specialized root structures and free-living rhizosphere bacteria, commonly referred to as plant growth-promoting rhizobacteria (PGPR) [9]. The broader term plant growth-promoting bacteria (PGPB) encompasses diverse taxa that enhance plant growth and yield through direct and indirect mechanisms [10,11]. Direct mechanisms include the facilitation of nutrient acquisition, such as phosphorus solubilization by genera including *Achromobacter*, *Pseudomonas*, and *Serratia* [12,13], as well as biological nitrogen fixation by genera such as *Bradyrhizobium*, *Sinorhizobium*/*Ensifer*, and *Mesorhizobium* [14]. Indirect mechanisms involve the suppression of soil phytopathogens [15] and the modulation of plant stress responses through enzyme activation and phytohormone regulation under saline conditions [16].

Numerous studies have demonstrated that PGPB inoculation can improve plant biomass, relative water content (RWC), and physiological performance under salt stress [17,18,19,20]. For example, beneficial effects on growth parameters and dry matter accumulation have been reported in cereals and horticultural crops under saline irrigation [18,19,20,21,22]. However, responses vary considerably depending on plant species, environmental conditions, inoculation timing, and strain composition. In some cases, synergistic effects among strains are not consistently observed, highlighting the complexity of plant–microbe–environment interactions.

Recent research suggests that multi-microbe applications in the form of microbial consortia may provide stronger or more stable effects than single-strain inoculations [23,24,25,26]. Functional complementarity among strains within a consortium may enhance nutrient acquisition, stress mitigation, and overall plant performance under saline conditions [24,25]. These findings support the hypothesis that consortium-based approaches, particularly those reflecting naturally structured microbial communities, may offer adaptive advantages over isolated strains.

In saline and desert ecosystems, long-term environmental filtering may select rhizosphere microbial communities intrinsically adapted to combined salinity and drought stress. Consequently, isolating indigenous PGPB from degraded or salt-affected soils has been proposed as an effective strategy for identifying functionally relevant microorganisms [27]. Nevertheless, it remains unclear whether a rhizosphere-derived indigenous consortium exhibits preferential effects on its original host species or whether its beneficial functions can be transferred across closely related plant species under saline irrigation.

To our knowledge, no previous study has evaluated whether a rhizosphere-derived indigenous bacterial consortium from *Lycium chinense* confers growth and physiological benefits to both its original host and a congeneric cultivated species under controlled saline irrigation.

Therefore, our research questions were:

(1) Can a bacterial consortium extracted from the rhizosphere of *Lycium chinense*, a salt-tolerant wild species native to Minqin Oasis NW China, promote plant growth under salt stress, in terms of biomass and physiological performance?
(2) Furthermore, can the above mentioned consortium, if it proves effective in promoting plant growth under saline conditions, be equally effective in promoting growth for a more widely used species in Europe, in our case *Lycium barbarum*?

## 2. Results

### 2.1. Identification of Inoculum Genera

**Figure 1:**
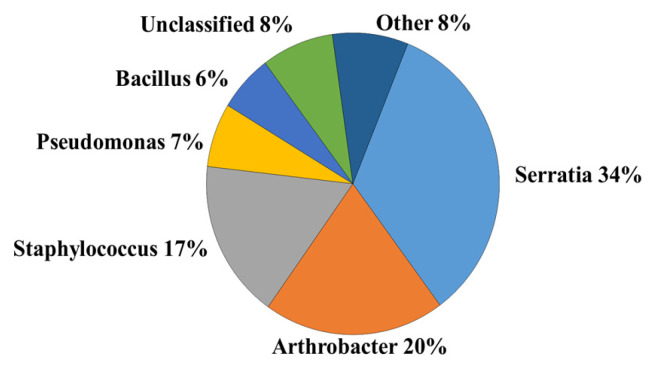
Percentage composition of the different bacterial genera present in the inoculum.

*Lycium chinense* soil rhizosphere was processed and characterized to obtain a bacteria culture to use in the experiments reported below. The genetic composition of the bacteria culture was assessed by applying a high-performance algorithm implemented in the GreenGenes taxonomic database of 16S rDNA, the gene encoding the 16S ribosomal RNA (rRNA) subunit found in bacteria and archaea. The composition of the bacterial genera consortium is reported in Figure 1 (see Section 4.2 for full details). The most abundant bacteria genus was *Serratia*, representing 34%, with the *S. entomophila* species (33%). Following *Serratia* was *Arthrobacter* (20%), including the two species *Artrobacter nicotianae* (12%) and *Arthrobacter soli* (2%). Then followed *Staphylococcus* with 17%, of which 9% was *Staphylococcus succinus*. Furthermore, *Pseudomonas* and *Bacillus* were the least abundant genera with 7% and 6%, respectively, and these two genera were present in *Pseudomonas panipatensis* (1.5%), *Pseudomonas chloritidismutans* (0.8%), and *Bacillus safensis* (0.54%) (Figure 1 and Data Sheet S1).

### 2.2. Overall Impact of Bacterial Inoculation on the Growth of Goji Plants Under Salt Stress

**Figure 2:**
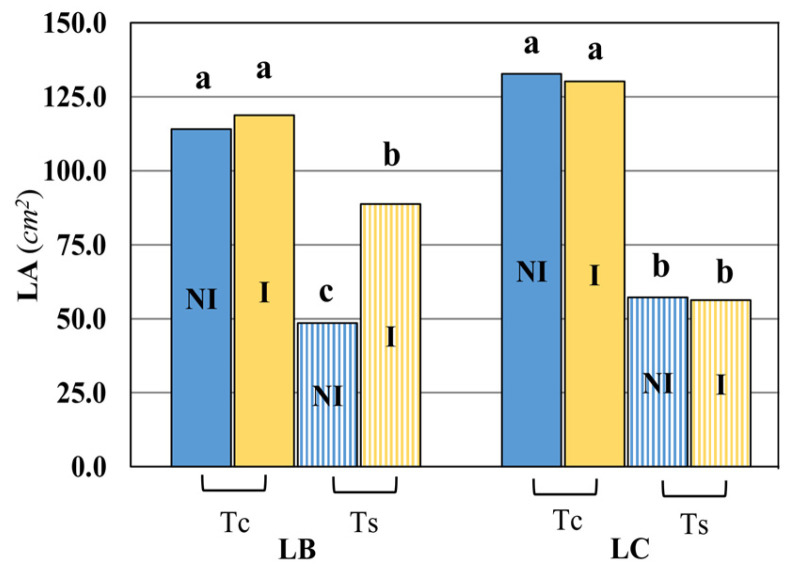
Impact of bacterial inoculation and salt stress on leaf area (LA) of *Lycium barbarum* (LB) and *Lycium chinense* (LC). Different letters indicate significant differences in means (n ≥ 8) at *p* < 0.05 according to the Duncan post hoc test. Tc, control irrigation; Ts, saline irrigation; I, consortium inoculation; NI, without consortium inoculation.

The leaf area (LA), under salinity conditions (Ts) and in response to bacteria inoculation (I), was evaluated in both *Lycium* species (Figure 2). No significant response of LA to bacteria was observed under non-saline irrigation of both LB (*Lycium barbarum*) and LC (*Lycium chinense*). Contrariwise, under saline irrigation (Ts), significant differences in LA were observed for LB but not for LC (Figure 2).

**Figure 3:**
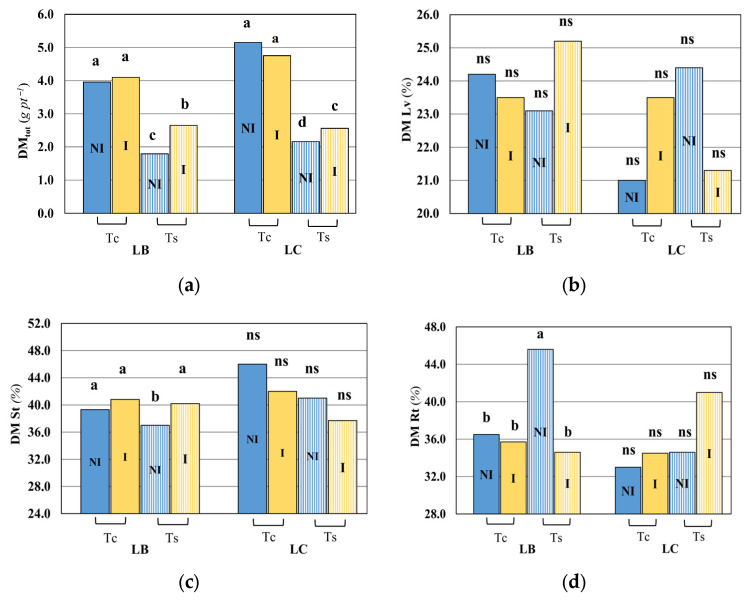
Impact of bacterial inoculation and salt stress on dry matter (DM) of *Lycium barbarum* (LB) and *Lycium chinense* (LC). The dry matter of leaves (DM Lv) (**b**), stem (DM St) (**c**), and roots (DM Rt) (**d**) is expressed as percentage of total dry matter (DM_tot_) (**a**). Different letters indicate significant differences in means (n ≥ 8) at *p* < 0.05 according to the Duncan post hoc test. Tc, control irrigation; Ts, saline irrigation; I, consortium inoculation; NI, without consortium inoculation.

The dry matter (DM) of different plant parts, as a percentage of the total dry matter, (Figure 3a–d) was generally consistent with the total dry matter (Figure 3a). The dry matter of leaves (DM Lv, Figure 3b) was not affected by salinity, and no impact of the consortium bacteria was found in both LB and LC.

Likewise, the difference in the stem’s dry matter (DM St) for LC was not significant under salinity conditions in response to the inoculation treatment (Figure 3c), compared with the control treatment (Tc) and the uninoculated (NI) ones. Contrariwise, the stem’s dry matter for LB under salinity conditions was significantly different in the bacteria-inoculated treatment compared to the uninoculated ones, while the control treatment did not yield any significant difference between the NI and I treatments. It should be noted that a similar response was found in root dry matter (DM Rt) in LB (Figure 3d). The overall difference between the NI and I treatments was significant under the saline conditions in LB for the leaf area, total dry matter, stem dry matter, and root dry matter, but not significant under the control conditions. No significant difference in root dry matter of LC was observed in all four treatments (Figure 3d).

**Figure 4:**
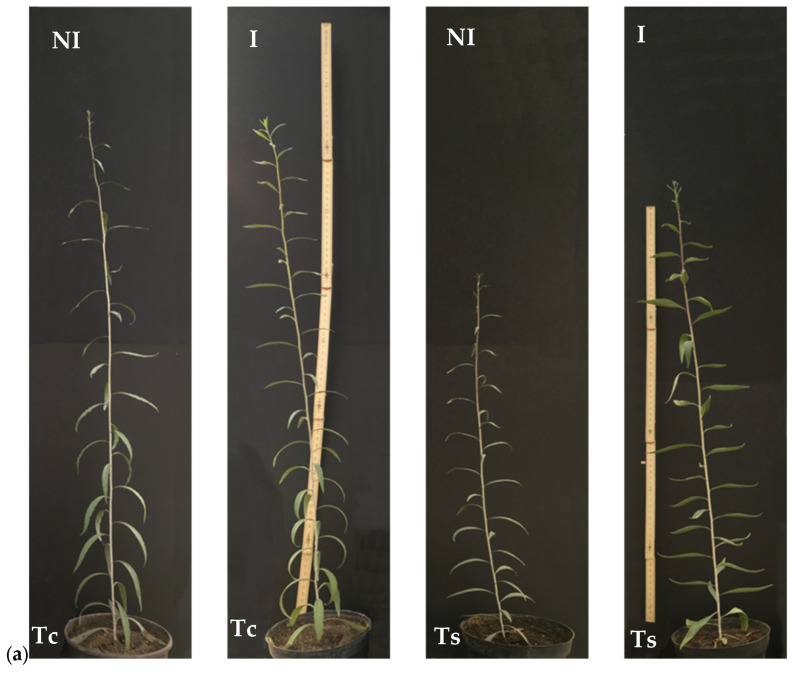

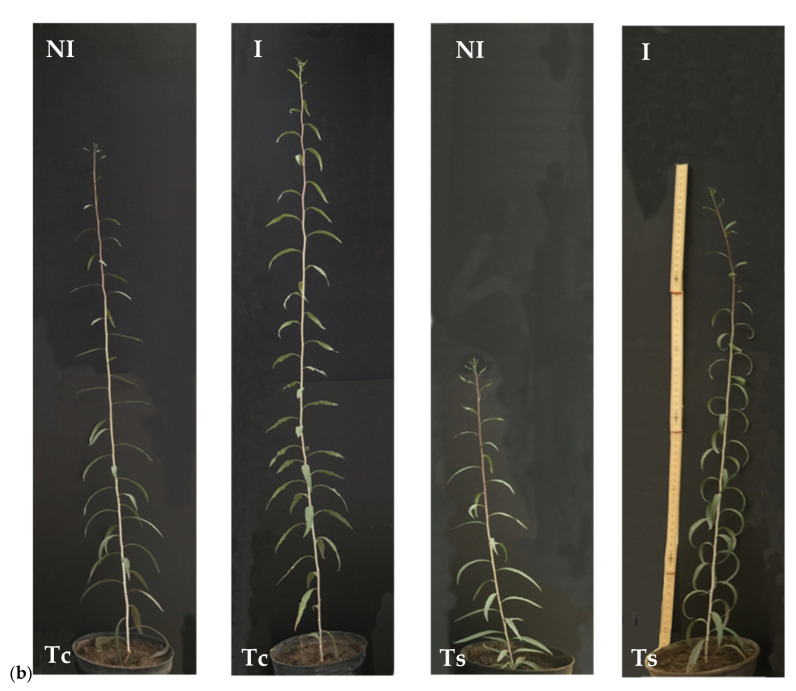
Phenotype of plants at the end of the experiment: 174 DAE for *Lycium barbarum* LB (**a**) and 181 DAE for *Lycium chinense* LC (**b**) in control (Tc) and salt stress (Ts) conditions, with (I) or without (NI) bacterial inoculation.

The total dry matter (DM tot) of both *Lycium* species decreased under saline irrigation compared with the control treatment, and under the uninoculated and inoculated treatments (Figure 3a). Moreover, in saline conditions the total dry matter was higher in response to the bacteria consortium treatment. The effects on the biomass and on crop growth are visible in Figure 4.

### 2.3. Physiological Responses to Bacterial Inoculation of Lycium Plants Under Salt Stress

**Figure 5:**
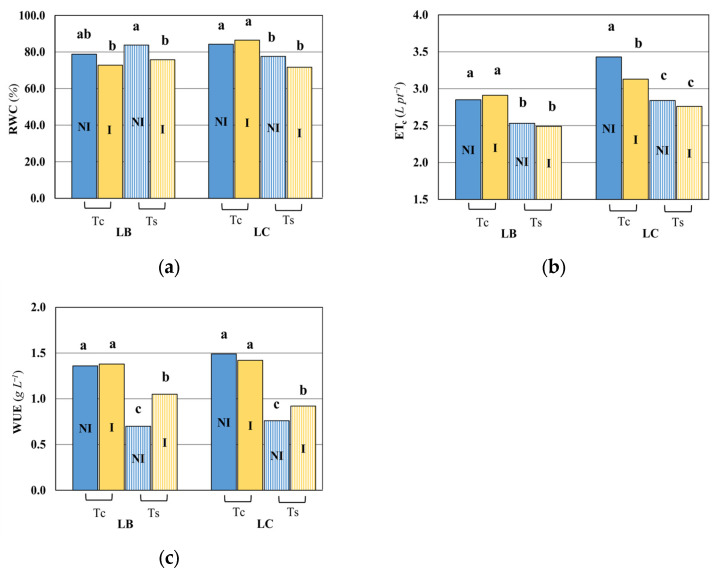
Impact of bacterial inoculation and salt stress on physiological parameters: relative water content (RWC) (**a**), evapotranspiration (ETc) (**b**) and water use efficiency (WUE) (**c**), measured in *Lycium barbarum* (LB) and *Lycium chinense* (LC). Different letters indicate significant differences in means (n ≥ 8) at *p* < 0.05 according to the Duncan post hoc test. Tc, control irrigation; Ts, saline irrigation; I, consortium inoculation; NI, without consortium inoculation.

The relative water content (RWC) was only reduced under salt stress in LC plants, while in LB the RWC was comparable in the Tc and Ts treatments with or without bacteria inoculum (Figure 5a). Contrariwise, the evapotranspiration (ETc) of both *Lycium* species was lower in the Ts treatment (Figure 5b). No impact of the bacteria treatment on the *Lycium barbarum* evapotranspiration was observed, while in *Lycium chinense* the evapotranspiration in the control treatment was higher, compared to the other thesis.

The water use efficiency (WUE) in both species under control conditions (Tc) was not significantly different between the uninoculated and inoculated treatments (Figure 5c). When salinity effect was considered (Ts), the WUE was significantly higher in the inoculated treatment. In detail, it led to higher a WUE in both LC and LB, with the increase in the latter being bigger: the WUE of LB under Ts increased from 0.7 to 1.05 in response to the bacteria treatment.

### 2.4. Detailed Analysis of the Response of LB and LC to the Treatment with Bacteria

As highlighted by the results illustrated in the figures of Section 2.2, the effects of inoculum and salinity are sometimes small and difficult to appreciate. Therefore, to further prove that salinity and inoculum affect the different parameters studied, the same data were processed, averaging by species (LB, LC), by non-saline (Tc) and saline water (Ts), and by the absence/presence of inoculum (NI, I).

In Table 1 no significant differences in dry matter (DM), water use efficiency (WUE), and leaf area (LA) were observed between LB and LC, while evapotranspiration (ETc) and relative water content (RWC) were higher in LC than LB at *p* ≤ 1% and *p* ≤ 5%, respectively. The higher water consumption of LC could be due to its 7-day longer cycle than LB during which it received two extra irrigations. All observed variables were lower under the Ts treatment at *p* ≤ 1%, except for RWC at *p* ≤ 5%.

Both dry matter and water use efficiency were significantly lower in response to the inoculated treatment at *p* ≤ 5% and *p* ≤ 1%, respectively. The inoculation (I) had a significant effect on relative water content (RWC); it was significantly higher under the inoculated treatment than the uninoculated ones, i.e., 82.5% vs. 77.0% at *p* ≤ 1%.

The analysis of several interactions gave other useful results, as shown in Table 2.

The *species* × *salinity* interaction shows that LC benefits most from the absence of salt for dry matter, relative water content, and water use efficiency, while the difference in evapotranspiration was not significant, despite presenting the same trend.

The *species* × *inoculum* interaction shows that LB responded positively and significantly to the inoculation with both a greater dry matter and a better use of water, as shown by the WUE values.

The *salinity* × *inoculum* interaction, significant for the dry matter and for the water use efficiency, highlights a positive effect of the inoculum in the presence of salt, while it had no effect in the case of irrigation with not-saline water.

To evaluate the effect of salinity on WUE, we estimated separate, specific equations (Table 3) for each cultivar and each salinity level in the presence and in the absence of bacteria, i.e., I vs. NI.

Therefore, for the same cultivar (LB) and the same water concentration (Tc), there is a significant difference in the slope *b* (*p* = 0.05) between the NI and I treatments. The bacteria treatment gave a better WUE under non-saline conditions (Tc) (*b*) than the uninoculated treatment, e.g., for an electrical conductivity in saturated paste (ECe) of 0.78 (dS m^−1^), the WUE in the I treatments is 0.77(g L^−1^) vs. 0.63 (g L^−1^) of NI treatment. For the same LB when salinity (Ts) is considered, there is a significant difference in the slope *b* that is, for the inoculated treatment, about twice the uninoculated thesis. It means that for the same ECe value (e.g., 6 dS m^−1^) the I treatment has a higher value of WUE than the value under NI treatment. It seems to be that the presence of bacteria with saline irrigation had a beneficial effect on the WUE.

Also, in the case of LC, the WUE increases with increasing ECe when the inoculum is applied. For example, for the same ECe the increase in WUE is higher in the I treatment compared to the NI treatments. The inoculation had a positive effect on the water use. Under salinity conditions (Ts), the inoculum determines a better performance in WUE. At the same ECe level in the I treatments, the WUE was higher than in the NI conditions.

**Figure 6:**
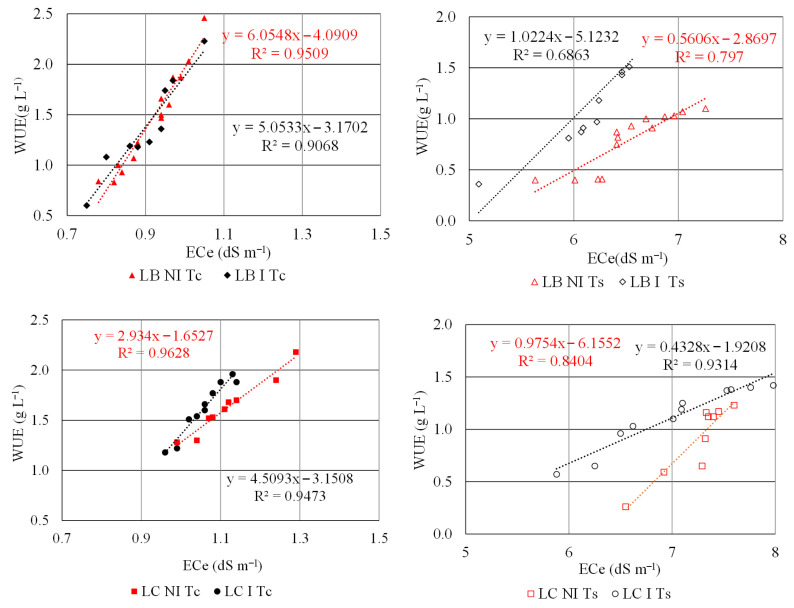
Linear regression of ECe against WUE for LB and LC species under Tc conditions in absence and presence of bacteria inoculum (NI vs. I) under non-saline water (Tc) and under salinity water (Ts).

The equations reported in Table 3 are also shown in Figure 6, where it is easier to see the increase in WUE at increasing electrical conductivity in saturated paste (ECe) for the dataset available.

Table 3 provides quantitative information, such as the significance and accuracy of the relationships which cannot be easily appreciated from the plots in Figure 6, which provide a qualitative and immediate overview of the WUE response to inoculation and salinity.

The trend is evaluated only within the range explored by the observations. Figure 5c describes the same results by considering the average WUE for the Ts treatments in the cases of LB and LC. Figure 6 shows all the observations available that were used to calculate the averages shown in Figure 5c.

## 3. Discussion

The results of our study highlight that the isolation of a bacterial consortium indigenous to a highly salinity environment had, a beneficial overall functional effect on plant function. Indeed, the bacterial consortium mitigated biomass reduction and improved WUE in plants inoculated under saline irrigation, while in treatments irrigated with non-saline water, the effect was limited. The significant *salinity* × *inoculum* interaction is indicative of the beneficial effect of the bacterial consortium, which clarifies the benefit of the inoculum in mitigating the effect of salt stress.

The PGPB consortium had a positive effect on relative water content (RWC).

The analysis of the interaction demonstrated that the beneficial effect of the inoculum was more evident on LB species. In LB there was a significant increase in DM for the *LB* × *I* interaction compared to *LB* × *NI* (3.38 vs. 2.87 g pt^−1^). The same response was observed for the WUE parameter (1.36 vs. 1.14 g L^−1^). For the same interaction (*species* × *inoculum*), the LC species did not show significant differences either for DM (3.6 vs. 3.67 g pt^−1^) or for WUE (1.31 vs. 1.23 g L^−1^) (Table 2). A likely explanation for these findings is that LC is already adapted to the bacteria consortium, which explains why the inoculation only had an impact on LB.

The results on the DM of LB were consistent with the ones observed by Shultana et al. [28] who observed a significant increase in rice DM treated with three different bacterial strains compared to the uninoculated saline control. Out of the three different bacterial strains, two of them belong to the *Bacillus* genus, the same genus present with 6% abundance in our consortium.

Further support is provided by the results of Ali et al. [29] who studied a Solanaceae relative of *Lycium*: tomato. They showed a significant increase in tomato DM under saline treatment and inoculated with two species of Pseudomonas (*P. migulae* and *P. fluorescens*), the same genus present in our consortium.

Upadhyay et al. [19,30] also tested several PGPB (mostly belonging to the Bacillus genus) on wheat under saline and non-saline conditions, finding significant increases in total biomass. Furthermore, focusing on the strains that gave a good improvement of wheat performance under saline conditions, they tested the combination of co-inoculation of some strains and found that, under saline conditions, the biomass significantly increased compared to both salinity controls and salinity with single strains, demonstrating that the combination of bacteria was better than with single inoculation.

Yasin et al. [31] studied the response of pepper plants in control and saline conditions. For both treatments, they evaluated the plant’s response after the application of bacterial inoculation, i.e., *Bacillus fortis* and *Pseudomonas aeruginosa*. Their results showed that the WUE of the saline treatment was 11% lower than the control, in uninoculated conditions. The trend observed in our experiment was similar to that seen in Yasin’s study, although the magnitude was rather different; in fact, the WUE for both *Lycium* species was 48% lower in the saline treatment than the control, in uninoculated conditions (Figure 5c). It was interesting to note that Yasin et al. also found that under saline conditions the WUE of the inoculated (*Bacillus Fortis*) treatment was 17% higher than the uninoculated treatment. A similar increase in WUE, i.e., 6%, was observed when comparing the treatment inoculated with *Pseudomonas aeruginosa* with the uninoculated treatment. The same genera were also present in our consortium; in fact, the WUE for the inoculated LB under saline irrigation was 50% higher than the uninoculated treatment. Results were qualitatively similar for LC with WUE being 21% higher than the uninoculated treatment. Moreover, pepper and *Lycium* belong to the same Solanaceae family. Inoculated plants were more hydrated than the control plants under saline conditions. These results demonstrated that the bacterial treatment efficiently protected the host plants against the detrimental effects of salt. We may speculate that the higher hydration induced by our bacteria consortium could be due to the enzymatic lowering of plant ethylene concentrations and improving WUE [32]. Nonetheless, our positive and preliminary results of the evaluation of the bacterial consortium on the biometric and physiological response of plants under saline conditions should be reconsidered in the light of further investigations relating to the individual bacterial strains and the evaluation of the enzymatic activities that the strains activate. Mayak et al. [20] recorded a significant increase in WUE in tomato plants inoculated under salt stress with *Achromobacter piechaudii* compared to uninoculated plants, i.e., 2.90 vs. 2.72 (mg biomass mg^−1^ H_2_O), showing a comparable change even though the WUE was determined on fresh biomass. Mayak et al. [20] also measured the phosphorus content, which was higher in inoculated plants. Evaluations of phosphorus on our plants have not been performed, but in the bacterial consortium used to inoculate our plants there is a high percentage (34%) of the Serratia genus which is known to have a great capacity in solubilizing insoluble forms of phosphorus in the soil, making it available to the plants [33,34,35]. Barra et al. [36] found that the Serratia genus was able to mitigate salinity stress by ACC (1-aminocyclopropane-1-carbossilato) deaminase production. We could speculate that the interaction and co-presence of different bacterial genera in our consortium had a beneficial effect on plants by improving their tolerance and performance under saline conditions [37,38].

Finally, the WUE when the *Species* × *Inoculation* and *salinity* × *inoculation* interactions were considered clearly increased in response to inoculation, as reported by Mayak et al. [20] and Yasin et al. [31].

If analyzed by the treatment and application of inoculum, it seems there were no significant differences in relative water content. Differences are found in the LC where relative water content was significantly different if we consider uninoculated *Lycium chinense* in control conditions (LC-NI-Tc) versus saline treatment (LC-NI-Ts) and when considering the inoculated ones (LC-I-Tc versus LC-I-Ts) (Figure 5a).

The contribution of inoculation to improve the hydration status (RWC) of plants in our study can be seen by analyzing the aggregated data in Table 1, where there was a significant difference between uninoculated (NI) and inoculated plants, i.e., 77% vs. 82%, suggesting that inoculation improves plant hydration. These results were in line with the findings of Shultana et al. [28] under salt stress conditions on rice plants treated with bacterial strains; they reported a significant increase in RWC compared to the uninoculated treatments, with RWC being significantly higher for all strains considered.

Of particular interest is the study of Nadeem et al. [39] which, under four salinity levels—where 5 dS m^−1^ is very close to our 6 dS m^−1^ treatment—found a significant increase in RWC in the saline inoculated treatment with strains W10 and W17, compared to the saline treatment without inoculation; the strains were respectively *Serratia* and *Pseudomonas*. These two genus strains are also present in our consortium. Also, Sapre et al. [40] reported a significantly higher RWC in plants under salinity when inoculated with *Klebsiella*, compared with the uninoculated one.

Considering the available literature on the mechanisms that bacteria activate to promote plant growth under stress conditions, we can speculate that the observed benefits on biomass, WUE, and RWC in our study are related to ACC deaminase activity, IAA (indole-3-acetic acid (IAA), and the solubilization of phosphorus and other nutrients.

The presence of *Serratia entomophila* (33%) and *Arthrobacter nicotianae* (12%) in the bacterial consortium, as highlighted in the literature, affects the solubilization of phosphorus and other nutrients under salt stress conditions. In the presence of salt stress, the above mentioned bacteria activate ACC deaminase, known to be the precursor of ethylene, which decreases under salt stress conditions, allowing greater hydration of the plants and thus improving salt tolerance [41,42,43]. In addition to the above mechanism, *Serratia* also activates indole-3-acetic acid (IAA), also activated by *Pseudomonas*, which enhances the length of lateral and adventitious roots, which in turn aid the host plant in maximizing its nutrient absorption.

The above mechanisms are also reported for other bacteria present in our consortium, namely: *Arthrobacter soli* (2%), *Staphilococcus succinus* (17%), *Pseudomonas panipatensis* (1.5%), *Pseudomonas chloritidismutans* (0.8%), and *Bacillus safensis* (0.54%) [43,44,45,46,47]. Therefore, although we did not monitor these IAA, ACC, and nutrient solubilization activities in our study, we can speculate that these were the mechanisms activated by the bacteria consortium to promote *Lycium* growth. After these initial findings, it is certainly necessary to evaluate the individual strains in terms of the activities they trigger and, based on the results obtained, the co-presence of targeted strains should be considered in a study on the possible amplification effect of co-inoculation.

Among the two *Lycium* species, the beneficial effect of inoculation with PGPB, in conditions of salt stress, was more evident in *L. barbarum* than in *L. chinense*. This more attenuated response of *L. chinense* may depend on several conditions:

(1) the PGPB consortium was isolated from the rhizosphere of LC already under saline conditions and therefore there could be a species/strain adaptation that mitigates the inoculation response on LC under saline conditions.

(2) among the two *Lycium* species, some authors reported the greater tolerance to salinity and drought of LC compared to LB [48,49] that partly explains the lower response of LC compared to LB.

The positive and improved response, in terms of biomass, water use efficiency, and relative water content, of the LB inoculated with PGPB consortium under saline conditions, was an initial positive finding that deserves further research aimed to assess the positive contribution of the identified bacteria species in PGPB activities.

Further research is also needed to assess whether indigenous consortia may function in a context-dependent and host-dependent manner, and that further strain-level and functional work is needed.

## 4. Materials and Methods

### 4.1. Site Description and Rhizospheric Soil Sampling

The study was carried out at the Minqin Drylands Salinization Research Station of the Key Laboratory of Deserts and Desertification, Northwest Institute of Eco-Environment and Resources, Chinese Academy of Science. The station, located in Xiquzhen, Gansu province in China (39°02′ N, 103°36′ E), was established to study the eco-environment of the area with the purpose of evaluating possible solutions against aridity and salinity. The Minqin Oasis, as well as the salinization research station, has a typical arid continental climate [50]. Due to the arid climate, agriculture depends completely on irrigation. In the surroundings of the station, several species survive the extreme conditions of soil salinity and water scarcity, such as halophytes, e.g., *Lycium chinense* (LC), with the common name Goji.

Rhizospheric soil of the halotolerant plant LC, from the saline desert of Xiquzhen, was collected during the end of the growing season in 2016 (July, summer) because during this period the LC plants were at full flowering stage. Rhizospheric soil from three replicate LC plants was collected by uprooting each plant with the root system and the soil attached to the roots was scraped using forceps. Microbial flora of a collected soil sample was analyzed within 4 days of the sample collection. Furthermore, soil samples were examined for physical and chemical properties. The soil characteristics at a soil depth of 0–20 cm of the study area are reported in Table 4.

### 4.2. Growth of Rhizosphere Bacteria, DNA Extraction and 16S Metagenomic Sequencing

The plant roots, after being cut into small pieces, were shacked for one hour in 10 mL of sterile 15 mM NaCl solution at room temperature to detach soil particles. After the decanting of the soil particles, the solution was employed for growing bacteria in Lauria–Bertani media (LB, Tryptone 10 gr/L, Yeast extract 5 gr/L, NaCl 10 gr/L) at 30 °C overnight. For the DNA extraction, 1 mL of bacteria culture was subjected to DNA extraction using the Wizard Genomic DNA Purification Kit (Promega Corporation 2800 Woods Hollow Road, Madison, WI 53711-5399 USA) following the manufacturer protocol. DNA quality control was performed by using a NanoDropOne spectrophotometer (Thermo Scientific, Waltham, MA, USA) and a Qubit Fluorometer 4.0 (Invitrogen Co., Carlsbad, CA, USA). PCR amplification was performed with primers: Forward: 5′-CCTACGGGNGGCWGCAG-3′; and Reverse: 5′-GACTACHVGGGTATCTAATCC-3′ [51], which target the hypervariable V3-V4 region of the 16S rRNA gene. Each PCR reaction was assembled according to 16S Metagenomic Sequencing Library Preparation (Illumina, San Diego, CA, USA). Libraries were quantified using the Qubit Fluorimeter (Invitrogen Co., Carlsbad, CA, USA) and pooled to an equimolar amount of each index-tagged sample to a final concentration of 4 mM, including the Phix Control Library (Illumina). Pooled samples were subject to cluster generation and sequenced on a MiSeq platform (Illumina, San Diego, CA, USA) in a 2 × 250 paired-end format at a final concentration of 10 pmol.

Next generation sequencing experiments, comprising quality control and primary bioinformatics analysis, were performed by Genomix4life S.R.L. (Baronissi, Salerno, Italy). The raw sequence files generated (fast files) underwent quality control analysis with FastQC.

### 4.3. Experiment Set Up and Measurements

**Figure 7:**
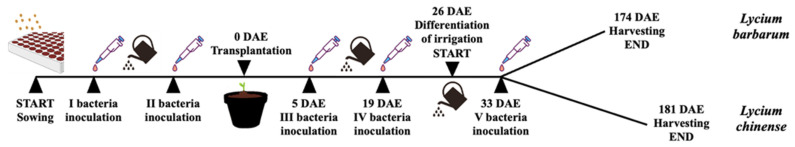
Experimental timeline from the seeds sowing to the harvesting of *Lycium barbarum* and *Lycium chinense*, where 0 DAE (day after emergence) corresponds to the transplantation day. Bacteria consortium inoculation is indicated by pipette and irrigations by watering can.

The experiment was carried out in a greenhouse. Climatic conditions during the experiment are presented in Figure S1. The species under study were *Lycium chinense* (LC) and *Lycium barbarum* (LB), the latter being more widespread in Europe, and it was decided to test the identified PGPB consortium on both LC and LB. The seeds of LC and LB were germinated in a plateau and one seedling was transferred into a pot on 10 November (0 DAE, day after emergence), (Figure 7).

The pots were filled with sterilized soil that had the following texture: 6.1% of clay, 17.7% of silt, 76.3% of sand, field capacity (FC) at −0.03 MPa 0.38 cm_w_^3^ cm_s_^−3^ (cubic cm of water on cubic cm of soil), and wilting point (WP) at −1.5 MPa 0.12 cm_w_^3^ cm_s_^−3^. Each pot had 16 cm diameter and 20 cm height, i.e., the soil volume in each pot was about 1800 cc. The experimental set up is summarized in Figure 7 and Figure S2 explains the experimental layout, where inoculated (I) means that five times during the growth cycle the bacteria consortium was added to the pot. The bacteria consortium was cultured and monitored through optical density measurements (OD 600 nm). At the exponential phase, the culture was diluted and poured on plates for single-colony counting; then, 1 mL at 10^7^ cfu/mL of the same bacteria culture was applied in the period in-between irrigations. The other treatment under study, reported as NI, means that no bacteria inoculation was applied to the plant. Two irrigation treatments were applied, non-saline water, which was our control treatment (Tc), and the saline water treatment (Ts). The treatments were replicated twelve times (4 pots × 3 replicates); therefore, for LC, a total of 48 pots were under study and the same number were under study for LB, so a total of 96 pots were monitored.

The differentiation of irrigation started on 6th December (26 DAE) (Figure 7). Saline water was produced by adding NaCl (56.8 mM), CaCl_2_ (0.76 mM), MgCl_2_ (3.4 mM), MgSO_4_ (3.07 mM) to the irrigation water. The composition of the saline irrigation water reproduces the composition of the groundwater in the oases of NW China. The electrical conductivity of the saline irrigation water was 6.8 dS m^−1^, whereas for the control water it was 1.0 dS m^−1^. Irrigation was applied when the control treatment (NI, Tc) reached the water content at wilting point (≅15 MPa) and we restored all treatments to the water content at a 0.03 MPa. The water consumption was monitored by weighing the pots at constant intervals. The registration of the water consumption allowed us to determine the crop evapotranspiration (ETc), since there was no precipitation. The plants were harvested: LB at 174 DAE and LC at 181 DAE, which received two more irrigations (Figure 7). At 174 DAE for LB and 181 DAE for LC, the number of leaves, the height, the leaf area, the relative water content, and the biomass were determined.

The plants in each pot were collected at harvest and the LA (cm^2^) was measured with a direct and destructive method by the LICOR-3100C leaf area meter (Li-COR Biosciences) at 174 DAE for LB and 181 DAE for LC, respectively. After that, the fresh weights of the leaves, stems, and roots were determined. To determine the total dry matter, the weighted materials were oven-dried at 65 °C until a constant weight was achieved to obtain the dry weight. Relative water content (RWC) was measured at harvest, and representative leaf samples were collected from each pot. The fresh weight of the leaves was recorded immediately after sampling to prevent moisture loss. Leaves were then floated in distilled water for 24 h at room temperature (about 15 °C) with no illumination to achieve full turgidity, after which excess surface moisture was gently removed, and the turgid weight was recorded. Finally, the leaves were oven-dried at 65 °C until a constant weight was achieved to obtain the dry weight (DW). RWC was calculated using the formula described by Barrs and Weatherley [52].

### 4.4. Statistical Analyses

Statistical analyses were performed on the basis of a split-plot design. Prior to the analysis of variance, the data on the observed variables (see Table 1) were grouped to generate samples by species, salinity level, and inoculation, and Bartlett’s test [53] was applied to assess the homogeneity of these samples. The variances were homogeneous; thus, the data were not transformed. The differences among treatments were assessed using the analysis of variance (ANOVA), followed by Duncan’s post hoc test to evaluate the significance of pairwise differences at a significance level of *p* < 0.05.

To evaluate certain interactions, e.g., species × salinity, the analysis of variance was performed by rearranging the observations according to a virtual split-plot design, in which the species were the main plot, and the saline concentration was the sub-plot, with the same approach applied for the other interactions. The dependence of WUE (g L^−1^) on ECe (dS m^−1^) was estimated by linear regression analyses.

## Figures and Tables

**Table 1:** Effects on crop evapotranspiration (ETc), dry matter (DM), water use efficiency (WUE), leaf area (LA), and relative water content (RWC). Paired values for cultivars, i.e., *Lycium Barbarum* (LB), *Lycium Chinense* (LC), water quality, i.e., non-saline (Tc) and saline water (Ts), and bacteria, i.e., no inoculation (NI) and inoculation (I), are averaged over the remaining four treatments.

Simple Effect	ETc (L pt^−1^)	DM tot (g pt^−1^)	WUE (g L^−1^)	LA (cm^2^)	RWC (%)
LB	2.42 ± 0.27 ^b^	3.13 ± 1.65 ^ns^	1.25 ± 0.55 ^ns^	95 ± 51.3 ^ns^	78.3 ± 5.8 ^B^
LC	2.76 ± 0.32 ^a^	3.63 ± 1.61 ^ns^	1.7 ± 0.45 ^ns^	94.4 ± 50.8 ^ns^	81.5 ± 7.7 ^A^
Tc	2.78 ± 0.31 ^a^	4.41 ± 1.50 ^a^	1.57 ± 0.42 ^a^	125.9 ± 45.5 ^a^	81.6 ± 7.2 ^A^
Ts	2.36 ± 0.21 ^b^	2.28 ± 0.97 ^b^	0.96 ± 0.39 ^b^	63.6 ± 34.8 ^b^	78.2 ± 6.3 ^B^
NI	2.55 ± 0.26 ^ns^	3.47 ± 1.50 ^A^	1.34 ± 0.48 ^a^	99.3 ± 51 ^ns^	77.0 ± 6.2 ^b^
I	2.67 ± 0.37 ^ns^	3.22 ± 1.78 ^B^	1.18 ± 0.54 ^b^	90.2 ± 50.8 ^ns^	82.5 ± 6.9 ^a^

Values followed by a different letter within a column are significantly different at the 1% (small letter) and 5% (capital letters) level of probability, ns = not significant according to Duncan’s test.

**Table 2:** Interaction effect of species (LB, LC) per salinity level (Tc, Ts) and per presence/absence of inoculum (I-NI).

Interaction Effects	ETc (L pt^−1^)	DM tot (g pt^−1^)	WUE (g L^−1^)	LA (cm^2^)	RWC (%)
Species × Salinity Concentration					
LB × Tc LB × Ts LC × Tc LC × Ts	2.6 ± 0.23 ^ns^ 2.23 ± 0.15 ^ns^	4.03 ± 1.63 ^B^ 2.22 ± 1.06 ^C^	1.52 ± 0.50 ^A^ 0.98 ± 0.45 ^B^	121.4 ± 49.0 ^ns^ 68.7 ± 38.4 ^ns^	76.4 ± 5.9 ^b^ 80.2 ± 5.4 ^b^
	3.00 ± 0.26 ^ns^ 2.53 ± 0.14 ^ns^	4.89 ± 1.14 ^A^ 2.36 ± 0.82 ^C^	1.62 ± 0.28 ^A^ 0.93 ± 0.29 ^B^	131.5 ± 39.8 ^ns^ 57.3 ± 28.5 ^ns^	86.6 ± 6.3 ^a^ 76.2 ± 7.0 ^b^
Species × Inoculation					
LB × I LB × NI LC × I LC × NI	2.42 ± 0.28 ^c^ 2.41 ± 0.25 ^c^ 2.67 ± 0.26 ^b^ 2.85 ± 0.34 ^a^	3.38 ± 1.59 ^A^ 2.87 ± 1.67 ^B^ 3.60 ± 1.37 ^A^ 3.67 ± 1.82 ^A^	1.36 ± 0.53 ^A^ 1.14 ± 0.55 ^B^ 1.31 ± 0.40 ^A^ 1.23 ± 0.49 ^A^	103.8 ± 49.8 ^ns^ 86.3 ± 51.3 ^ns^ 93.8 ± 51.8 ^ns^ 95.0 ± 49.6 ^ns^	74.6 ± 4.3 ^ns^ 82.0 ± 6.0 ^ns^ 80.0 ± 7.4 ^ns^ 83.3 ± 7.8 ^ns^
Salinity Concentration × Inoculation					
Ts × I Ts × NI Tc × I Tc × NI	2.34 ± 0.21 ^ns^ 2.39 ± 0.21 ^ns^ 2.73 ± 0.24 ^ns^ 2.82 ± 0.37 ^ns^	2.61 ± 1.05 ^b^ 1.95 ± 0.74 ^c^ 4.34 ± 1.38 ^a^ 4.49 ± 1.60 ^a^	1.11 ± 0.42 ^b^ 0.8 ± 0.28 ^c^ 1.57 ± 0.41 ^a^ 1.56 ± 0.43 ^a^	74.8 ± 39.5 ^ns^ 52.4 ± 24.6 ^ns^ 123.9 ± 49.3 ^ns^ 127.9 ± 41.2 ^ns^	74.3 ± 4.6 ^ns^ 82.4 ± 6.5 ^ns^ 79.7 ± 7.0 ^ns^ 82.7 ± 7.2 ^ns^

Values followed by a different letter within a column are significantly different at the 1% (small letters) and 5% (capital letters) level of probability, and ns = not significant according to Duncan’s test.

**Table 3:** Relationship between water use efficiency (WUE, g L^−1^) and ECe (electrical conductivity in saturated paste, dS m^−1^) for the different cultivars (LB, LC) irrigated by non-saline (Tc) and saline water (Ts) in the presence of inoculation (I) or absence of inoculation (NI) (fd = freedom degree).

Treatment	Equations	fd	R^2^	Significance Test of Slopes b
LB-NI-Tc	WUE = 6.05 × ECe − 4.09	19	0.95	1.94 *
LB-I-Tc	WUE = 5.05 × ECe − 3.17		0.91	
LB-NI-Ts	WUE = 0.561 × ECe − 2.87	20	0.80	1.85 *
LB-I-Ts	WUE = 1.02 × ECe − 5.12		0.69	
LC-NI-Tc	WUE = 2.93 × ECe − 1.65	16	0.96	
LC-I-Tc	WUE = 4.51 × ECe − 3.15		0.94	3.28 **
LC-NI-Ts	WUE = 0.97 × ECe − 6.15	15	0.84	
LC-I-Ts	WUE = 0.43 × ECe − 1.92		0.93	3.50 **

* Significant per *p* ≤ 0.05; ** significant per *p* ≤ 0.01.

**Table 4:** Soil characteristics at soil depth of 0–20 cm of the study area.

Parameters	Unit	Soil Layer 0–20 cm
Clay	%	7.00
Silt	%	44.3
Sand	%	48.7
Bulk density	g cm^−3^	1.40
pH	-	7.5
Ece	dS m^−1^	2.0
Ca^2+^	g kg^−1^	0.053
Mg^2+^	g kg^−1^	0.031
K^+^	g kg^−1^	0.016
Na^+^	g kg^−1^	0.032
N total	g kg^−1^	0.757
Total Carbon	g kg^−1^	17.3
Available P	mg kg^−1^	15.58

## Data Availability

The data presented in this study are available upon request from the corresponding author.

## Supplementary Materials

The following supporting information can be downloaded at https://www.mdpi.com/article/10.3390/plants15060892/s1: Figure S1, Climatic trend during the experiment; Figure S2, Experimental set-up with the different treatments and replicate numbers; Data Sheet S1, Detailed species inside the bacterial consortium.
