# Supplementary material for: Are Autochthonous Bacteria of Desert Root Environments Capable of Increasing Crop Tolerance to Saline Stress?
Source: Plants (Basel). 2026 Mar 13;15(6):892. doi: 10.3390/plants15060892 (PMC13030662; doi:10.3390/plants15060892)
Supplement: Supplementary file 1 [file plants-15-00892-s001.zip › Fig-S1-S2.pdf]

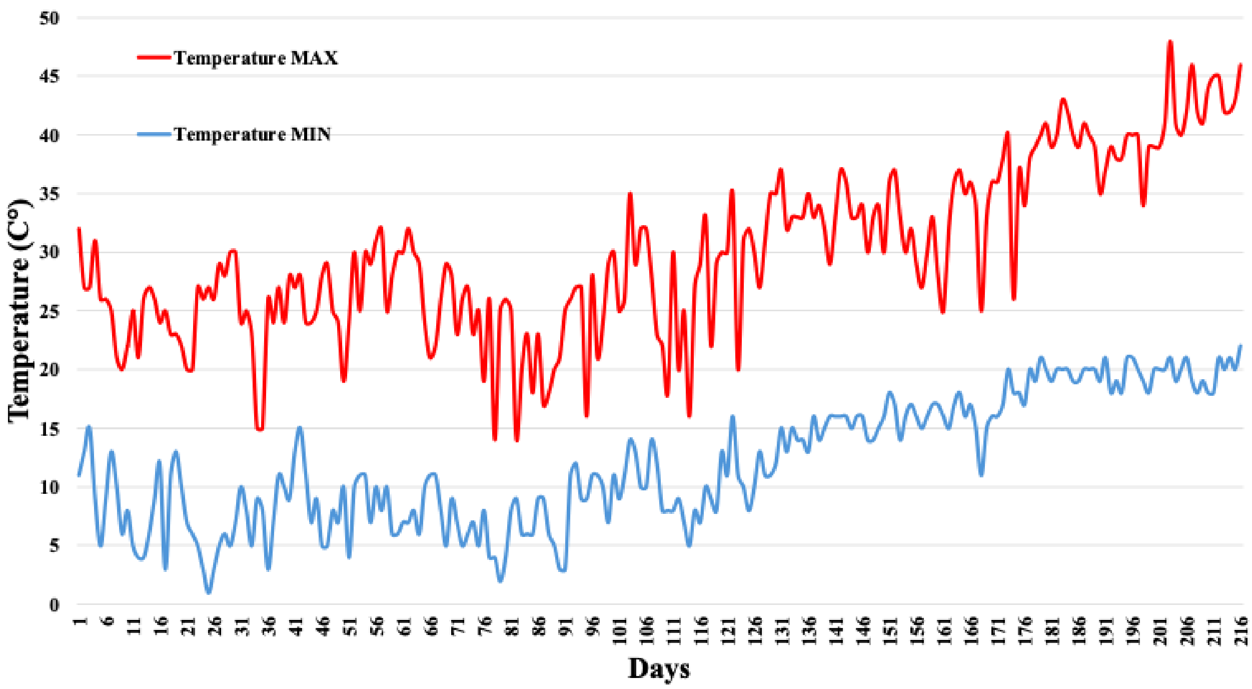

Figure S1 Climatic trend during the experiment with minimum (Temperature MIN) and maximum (Temperature MAX) temperature.

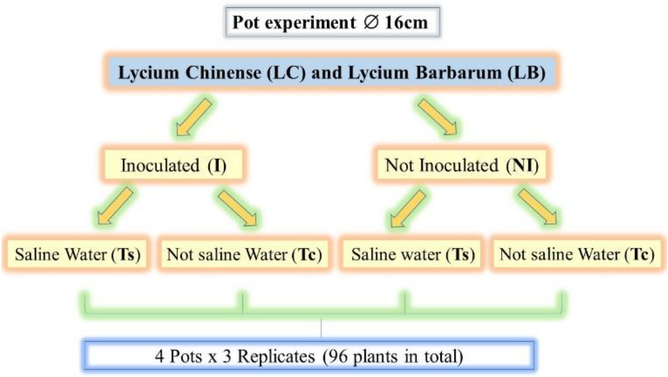

Figure S2. Experimental set-up with the different treatments and replicate numbers.
